# Interpreting economic complexity

**DOI:** 10.1126/sciadv.aau1705

**Published:** 2019-01-09

**Authors:** Penny Mealy, J. Doyne Farmer, Alexander Teytelboym

**Affiliations:** 1 Institute for New Economic Thinking at the Oxford Martin School, University of Oxford, Oxford OX2 6ED, UK.; 2 Smith School for Enterprise and the Environment, University of Oxford, Oxford OX1 3LP, UK.; 3 Bennett Institute for Public Policy, University of Cambridge, Cambridge, CB3 9DT, UK.; 4 Department of Economics, University of Oxford, Oxford OX1 3UQ, UK.; 5 Department of Computer Science, University of Oxford, Oxford OX1 3QD, UK.; 6 Santa Fe Institute, Santa Fe, NM 87501, USA.; 7 Mathematical Institute, University of Oxford, Oxford OX1 3LP, UK.

## Abstract

Two network measures known as the economic complexity index (ECI) and product complexity index (PCI) have provided important insights into patterns of economic development. We show that the ECI and PCI are equivalent to a spectral clustering algorithm that partitions a similarity graph into two parts. The measures are also closely related to various dimensionality reduction methods, such as diffusion maps and correspondence analysis. Our results shed new light on the ECI’s empirical success in explaining cross-country differences in gross domestic product per capita and economic growth, which is often linked to the diversity of country export baskets. In fact, countries with high (low) ECI tend to specialize in high-PCI (low-PCI) products. We also find that the ECI and PCI uncover specialization patterns across U.S. states and U.K. regions.

## Teaser

Beyond diversity, towards similarity: Math of economic complexity uncovers specialization patterns across countries and regions.

## INTRODUCTION

Structural properties of the global trade network can explain differences in economic development across countries (*1*–*6*). A novel pair of measures known as the economic complexity index (ECI) and the product complexity index (PCI) were recently introduced to infer information about countries’ productive capabilities from their export baskets (*2*, *3*). These measures have been particularly successful in explaining cross-country differences in gross domestic product (GDP) per capita and in predicting economic growth. However, the precise mathematical and economic interpretations of these indices have been elusive.

Here, we show that the economic complexity measures are mathematically equivalent to a classic spectral clustering algorithm, which partitions a similarity graph into two balanced components that are internally similar and externally dissimilar (*7*). The ECI and PCI can also be interpreted as dimensionality reduction methods, which have close connections to diffusion maps (*8*) and correspondence analysis (*9*–*14*). These approaches have already been used in many disciplines, including archaeology, ecology, and engineering (*15*).

We offer two interpretations of the ECI and PCI from a dimensionality reduction perspective. First, the ECI and PCI measures can be seen as defining a distance between nodes in a graph on the basis of their similarity. Consequently, when applied to export data, the ECI (PCI) places countries (products) on a one-dimensional interval such that countries (products) with similar exports (exporters) are close together and countries (products) with dissimilar exports (exporters) are far apart. Second, the ECI and PCI can be interpreted as orderings that maximize the correlation between two categorical variables.

Our mathematical interpretations contrast previous conceptual descriptions of the economic complexity measures, which tended to frame the ECI as being related to the diversity (or number) of products a country is able to export competitively (*2*, *3*, *16*, *17*). Not only is the ECI mathematically orthogonal to diversity (*18*), but, as we show, it also captures insightful information that diversity does not make apparent. When applied to export data, the ECI and PCI reveal a striking pattern of specialization across countries. High-ECI countries (which tend to be richer) specialize in high-PCI products [which tend to be more technologically sophisticated (*2*)]. Countries with low ECI (which tend to be poorer) specialize in low-PCI products (which tend to be less technologically sophisticated). Moreover, the export baskets of high-ECI countries are more homogeneous than the export baskets of low-ECI countries. Hence, while diversity counts how many products countries are competitive in, the ECI and PCI help distinguish products that high- and low-income countries specialize in.

Our results also allow us to extend the ECI and PCI to datasets other than trade data. We provide an illustration with regional data on industrial employment concentrations in U.K. local authorities and occupational employment concentrations in U.S. states. We find that, remarkably, the ECI for U.K. local authorities and U.S. states is strongly correlated with regional earnings per capita. We also show that the ECI and PCI reveal similar patterns of specialization, while diversity fails to be economically informative.

## THE ECI AND PCI

The ECI and PCI measures are calculated using an algorithm that operates on a binary country-product matrix *M* with elements *M*_*cp*_, indexed by country *c* and product *p* (*3*). *M*_*cp*_ = 1 if country *c* has a revealed comparative advantage (RCA) > 1 in product *p*, where RCA is calculated using the Balassa index (*19*), given by

$${\text{RCA}}_{cp}=\frac{x_{cp}/\Sigma_{p}x_{cp}}{\underset{c}{\Sigma }x_{cp}/\Sigma_{c}\Sigma_{p}x_{cp}}$$ (1)

where *x*_*cp*_ is country *c*’s exports of product *p*. *M*_*cp*_ = 0 otherwise. If *M*_*cp*_ = 1, we say that country *c* is competitive in product *p*.

Summing across the rows and columns of *M* gives a country’s diversity [denoted $k_{c}^{\left(0\right)}$] and product ubiquity [denoted $k_{p}^{\left(0\right)}$], defined as

$$k_{c}^{\left(0\right)}=\underset{p}{\Sigma }M_{cp}$$ (2)

and

$$k_{p}^{\left(0\right)}=\underset{c}{\Sigma }M_{cp}$$ (3)

The ECI and PCI were originally defined through an iterative, self-referential method of reflections algorithm that first calculates diversity and ubiquity and then recursively uses the information in one to correct the other (*3*). However, it can be shown (*1*, *20*) that the method of reflections is equivalent to finding the eigenvalues of a matrix $\tilde{M}$, whose rows and columns correspond to countries and whose entries are given by

$${\tilde{M}}_{cc\prime }\equiv \underset{p}{\Sigma }\frac{M_{cp}M_{c\prime p}}{k_{c}^{\left(0\right)}k_{p}^{\left(0\right)}}=\frac{1}{k_{c}^{\left(0\right)}}\underset{p}{\Sigma }\frac{M_{cp}M_{c\prime p}}{k_{p}^{\left(0\right)}}$$ (4)

Equivalently, we can write $\tilde{M}$ in matrix notation

$$\tilde{M}=D^{-1}MU^{-1}M\prime$$ (5)

where *D* is the diagonal matrix formed from the vector of country diversity values and *U* is the diagonal matrix formed from the vector of product ubiquity values.

When applied to country trade data, one can think of $\tilde{M}$ as a diversity-weighted (or normalized) similarity matrix, reflecting how similar two countries’ export baskets are.

Further, from Eq. 5, we can see that

$$\tilde{M}=D^{-1}S$$ (6)

where *S* = *MU*^−1^*M*′ is a symmetric similarity matrix in which each element $S_{cc\prime }$ represents the products that country *c* has in common with country *c*′, weighted by the inverse of each product’s ubiquity.

Since $\tilde{M}$ is a row-stochastic matrix (its rows sum to one), its entries can also be interpreted as conditional transition probabilities in a Markov transition matrix (*3*, *18*). The ECI is defined as the eigenvector associated with the second largest right eigenvalue of $\tilde{M}$. This eigenvector determines a “diffusion distance” between the stationary probabilities of states reached by a random walk described by this Markov transition matrix (see the “Diffusion map distance” section and the Supplementary Materials).

The PCI is symmetrically defined by transposing the country-product matrix *M* and finding the eigenvector corresponding to the second largest right eigenvalue of $\hat{M}$, given by

$$\hat{M}=U^{-1}M\prime D^{-1}M$$ (7)

Here, we denote the ECI vector by ${\tilde{y}}^{\left[2\right]}$ and the ECI of country *c* is denoted ${\tilde{y}}_{c}^{\left[2\right]}$. We also denote the diversity vector by *d*, where $d_{c}=k_{c}^{\left(0\right)}$ is the diversity of country *c*. In addition, we note that the ECI is commonly standardized by subtracting the mean and dividing the difference by the SD of ${\tilde{y}}^{\left[2\right]}$ to allow for comparisons across years (*2*, *3*). However, for clarity, we use the unstandardized ECI vector throughout this paper.

## RESULTS

The ECI has commonly been described with reference to diversity. This follows from the hypothesis that originally motivated the measure’s construction: Prosperous countries are likely to be able to competitively export a diverse set of products that few other countries are competitive in (*2*, *3*). Recent papers have since described the ECI as an “indicator of diversity” [(*17*), p. 1] and a “measure of economic diversity” [(*16*), p. 1596]. However, the ECI has been shown to be mathematically orthogonal to diversity (*18*). That is, the dot product of the diversity and ECI vectors is zero.

The ECI has also been described as a “standard eigenvalue centrality algorithm” [(*17*), p. 1]. However, this description is also inaccurate, as in contrast to the ECI, eigenvector centrality is defined as the eigenvector corresponding to the largest eigenvalue of a symmetric adjacency matrix, such as *S*. In the case of directed networks (such as $\tilde{M}$), since the right eigenvector corresponding to the largest eigenvalue is constant, the natural definition would take the left eigenvector corresponding to the largest eigenvalue of the adjacency matrix [(*21*), p. 178]. [Note that, in the exposition of (*21*), adjacency matrices are transposed.] Moreover, since the rows of $\tilde{M}$ have been normalized by diversity, the leading left eigenvector (eigenvector centrality) will be proportional to diversity and consequently does not add any information about $\tilde{M}$.

### Interpretation as spectral clustering

We now show that the ECI is mathematically equivalent to a standard spectral clustering method for partitioning an undirected weighted graph, represented by an adjacency matrix *S*, into two balanced components (*7*). Spectral clustering is a widely used technique for community detection and dimensionality reduction and has a range of applications including image recognition, web page ranking, information retrieval, and RNA motif classification. The goal of one spectral clustering approach is to minimize the sum of the edge weights cutting across the graph partition, while making the size (number of nodes) of the two components relatively similar [also known as the normalized cut (Ncut) criterion] (*7*). As we discuss below, finding the exact solution to this problem is NP-hard. However, it is possible to obtain an approximate solution (*7*). We demonstrate that the ECI is equivalent to this approximate solution.

#### The Ncut criterion

Consider an undirected graph *G* = (*V*, *E*) with vertices *V* and edges *E*. We allow the graph *G* to be weighted with nonnegative weights; thus, the adjacency matrix entries are *S*_*ij*_ ≥ 0, where *S*_*ij*_ = *S*_*ji*_. While the export matrix is one possible example, we can consider *S* to be any similarity or affinity matrix with these properties. The degree of vertex *i* is defined as

$$d_{i}=\underset{j\in V}{\Sigma }S_{ij}$$ (8)

and the size or “volume” of a set of vertices *A* ⊆ *V* can be measured as

$$\text{vol}(A)=\underset{i\in A}{\Sigma }d_{i}$$ (9)

Our notation is deliberate: As we show in the Supplementary Materials, if the adjacency matrix *S* of the similarity graph *G* coincides with export similarity matrix $S=D\tilde{M}$, then degree *d*_*i*_ corresponds precisely to the diversity of a country’s exports.

One way to partition a graph into two disjoint sets is by solving the cut problem. The objective is to find a partition of *V* into complementary sets *A* and $\bar{A}$ that minimizes the number of links between the two sets. The cut problem is to find the minimum of

$$\text{cut}(A,\bar{A})=\underset{i\in A,j\in \bar{A}}{\Sigma }S_{ij}$$ (10)

This objective function has the undesirable property that its minimum often partitions a single node from the rest of the graph. To avoid this problem, the Ncut criterion (*7*) penalizes solutions that are not properly balanced. The objective is to partition the graph in such a way that each cluster contains a reasonable number of vertices. This can be achieved by minimizing the objective function

$$\text{Ncut}(A,\bar{A})=(\frac{1}{\text{vol}(A)}+\frac{1}{\text{vol}(\bar{A})})\underset{i\in A,j\in \bar{A}}{\Sigma }S_{ij}$$ (11)

Let *D* be the diagonal degree matrix with *D*_*ii*_ = *d*_*i*_ and *D*_*i* ≠ *j*_ = 0. Then, finding the minimum value of Ncut is equivalent to solving the optimization problem

$$\underset{A}{\text{min}}\;\text{Ncut}(A,\bar{A})=\underset{y}{\text{min}}\frac{y^{T}(D-S)y}{y^{T}Dy}$$ (12)

subject to *y*_*i*_ ∈ {1, – vol(*A*)/vol($\bar{A}$)} and *y*^*T*^*D***1** = 0.

Because *y*_*i*_ is restricted to one of two possible values, this is not a simple linear algebra problem, and finding the true minimum of the Ncut criterion has been shown to be NP-hard (*7*). However, by letting *y*_*i*_ take on any real value, an approximate solution can be obtained by finding the eigenvector *y*^[2]^ corresponding to the second smallest eigenvalue of the generalized eigenvalue equation

(D−S)y=λDy (13)

Recall that *L*_*S*_ = *D* – *S* is called the Laplacian matrix of *S*. By making the substitution

$$y=D^{-1/2}z$$ (14)

this can be rewritten as a standard eigenvalue equation

$$D^{-\frac{1}{2}}(D-S)D^{-\frac{1}{2}}z={\bar{L}}_{S}z=\lambda z$$ (15)

where ${\bar{L}}_{S}=D^{-\frac{1}{2}}(D-S)D^{-\frac{1}{2}}$ is the normalized Laplacian of *S*. Because the normalized Laplacian is a stochastic matrix, its smallest eigenvalue is zero. The eigenvector *z*^[2]^ associated with the second smallest eigenvalue of ${\bar{L}}_{S}$ is called the normalized Fiedler vector and is a solution to the standard eigenvalue equation in Eq. 15. Transforming back to *y* using Eq. 14 to solve the original problem gives the solution

$$y^{\left[2\right]}=D^{-1/2}z^{\left[2\right]}$$ (16)

The solution *y*^[2]^ provides a useful approximate solution to minimizing the Ncut criterion and is equal to a simple transformation of the normalized Fiedler vector (*7*).

#### The relationship between the ECI and the Ncut criterion

Recall that $\tilde{M}$ is the matrix whose eigenvector corresponding to the second largest eigenvalue is the ECI. To see the relationship between spectral clustering and the ECI, note that the similarity matrix $S=D\tilde{M}$ characterizing country export similarity is in the same form used to minimize the Ncut criterion. Multiplying both sides of Eq. 15 by $D^{-\frac{1}{2}}$ and rearranging terms give

$$D^{-1}SD^{-\frac{1}{2}}z=(1-\lambda )D^{-\frac{1}{2}}z$$ (17)

Substituting $\tilde{M}=D^{-1}S$ gives

$$\tilde{M}D^{-\frac{1}{2}}z=(1-\lambda )D^{-\frac{1}{2}}z$$ (18)

The eigenvalue equation for $\tilde{M}$ is

$$\tilde{M}\tilde{y}=\tilde{\lambda }\tilde{y}$$ (19)

Now, comparing Eqs. 18 and 19, we can see that the eigenvalues and eigenvectors of $\tilde{M}$ are related to those of ${\bar{L}}_{S}$ by

$$\tilde{\lambda }=1-\lambda$$ (20)

and

$$\tilde{y}=D^{-\frac{1}{2}}z$$ (21)

Thus, the second smallest eigenvalue of ${\bar{L}}_{S}$ corresponds to the second largest eigenvalue of $\tilde{M}$, and comparison to Eq. 16 makes it clear that the ECI is equivalent to approximately minimizing the Ncut criterion, that is

$${\tilde{y}}^{\left[2\right]}=y^{\left[2\right]}=D^{-\frac{1}{2}}z^{\left[2\right]}$$ (22)

where ${\tilde{y}}^{\left[2\right]}$ represents the second largest eigenvector of $\tilde{M}$.

This implies that the ECI (${\tilde{y}}^{\left[2\right]}$) is equivalent to the approximate solution (*y*^[2]^) that minimizes the Ncut criterion on the matrix *S*. Moreover, the ECI is related to the normalized Fiedler vector by a simple transformation. In the Supplementary Materials, we also show how this interpretation can be applied to the PCI and describe the mathematical relationship between the ECI and PCI.

#### Applying the spectral clustering interpretation to economic data

**Fig. 1:**
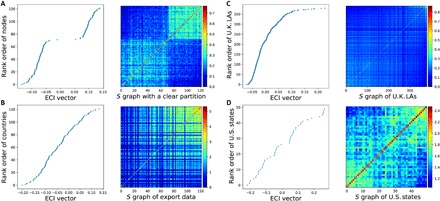
Interpreting the ECI as a spectral clustering method. Each panel shows the ECI vector (in ascending order) (left) and the associated similarity matrix *S* (right), where rows and columns have been ordered by the ECI and colored by the *S*_*ij*_ values. Panels correspond to similarity networks based on (**A**) randomly generated data with two clear components, (**B**) HS6 COMTRADE data for 2013, (**C**) data on employment concentrations in different industries in U.K. local authorities (LAs), and (**D**) data on employment concentrations in different occupations in U.S. states.

We now demonstrate how the ECI partitions similarity networks in practice. A visual illustration is shown in Fig. 1A. Here, we have calculated the ECI for a randomly generated similarity graph with two clear components. The ECI assigns each node a real number on an interval with positive and negative values according to their similarity to each other. In Fig. 1A (left), we show the ECI values associated with each node in ascending order. The graph should be partitioned where ECI is zero. Nodes with a positive ECI are assigned to one cluster, and nodes with a negative ECI go into the other cluster. In this case, the distinct gap in the ECI values shows that the partition is very clear. In Fig. 1A (right), we show the network’s adjacency matrix *S*, where we have also ordered the rows and columns in accordance with the ascending ECI values. Here, one can also see how the ECI ordering reveals the graph’s two clear components.

In Fig. 1B, we show the same for export data (based on HS6 COMTRADE data for the year 2013). In Fig. 1B (left), country ECI values (sorted in ascending order) do not show a clear gap across the zero threshold. Moreover, Fig. 1B (right) suggests that, while countries with high ECI values have a high degree of similarity in their exports (as shown by the higher *S*_*ij*_ values), countries with low ECI values appear to have more varied export portfolios. These plots therefore indicate that the export data do not partition clearly into two components.

In Fig. 1 (C and D), we apply the ECI to two other similarity networks constructed from regional data for the United Kingdom and the United States. Figure 1C shows a similarity graph constructed on the basis of regional data from the U.K. Business Register and Employment Survey (BRES) for the year 2011 (available from www.nomisweb.co.uk/). Here, nodes are U.K. local authorities, which are similar to each other on the basis of their employment concentrations in different industries (classified at the three-digit level of granularity). The similarity graph in Fig. 1D is constructed from regional data sourced from the Integrated Public Use Microdata Series (IPUMS) (*22*) for the year 2010 (available from https://usa.ipums.org/usa/). In this graph, nodes are U.S. states, and similarity is calculated on the basis of employment concentrations in different occupations (also classified at the three-digit level of granularity). More details about the construction of these networks can be found in Materials and Methods.

In both of these examples, the data do not partition clearly into two components either (further analysis using the eigengap heuristic can be found in the Supplementary Materials). However, as we show in the next section, the ECI and PCI nonetheless glean useful information from economic datasets.

### Interpretations as dimensionality reduction tools

In addition to approximating the Ncut criterion, the economic complexity measures can also be interpreted as dimensionality reduction tools. We discuss two such interpretations.

#### Diffusion map distance

The first interpretation comes from Shi and Malik’s (*7*) observation that the ECI exactly minimizes

$$\frac{\Sigma_{ij}{\left(y_{i}-y_{j}\right)}^{2}S_{ij}}{\Sigma_{i}y_{i}^{2}d_{i}}$$ (23)

subject to the constraint

$$\underset{i}{\Sigma }y_{i}d_{i}=0$$ (24)

Here, the objective is to find real numbers *y*_*i*_ for each node *i* that minimize the sum of the squared distances between nodes, where the distances are weighted according to the similarity matrix *S*. The constraint ensures that the assigned numbers *y*_*i*_ take on positive and negative values and are reasonably balanced in their distribution above and below zero. As we will discuss further in the “Revisiting previous interpretations of economic complexity” section, it also hard-wires the orthogonality condition between the ECI and diversity vectors.

When applied to export data, we can interpret the ECI as a method to collapse the high-dimensional space of country-export similarities into one dimension. The ECI positions countries on an interval where similar countries are placed close together and dissimilar countries are placed far apart. The distance between countries on this line is a special case of the “diffusion map distance” (*8*), which we discuss further in the Supplementary Materials.

**Fig. 2:**
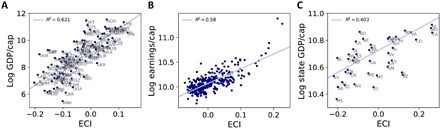
ECI versus income per capita. (**A**) Relationship between the ECI and log GDP per capita for data on countries and exports. (**B**) Relationship between the ECI and log per-capita earnings for data on industrial employment concentrations in U.K. local authorities. As the scatterplot is too tightly clustered to show legible local authority labels, we provide the top and bottom 10 local authorities ranked by their ECI in the Supplementary Materials. (**C**) Relationship between the ECI and log GDP per capita for data on occupational employment concentrations in U.S. states.

What makes this interpretation interesting from an economic perspective is the fact that the ECI correlates strongly with per-capita GDP (see Fig. 2A) (*2*, *3*). It is not immediately obvious that placing countries along a monodimensional continuum on the basis of the similarity of their exports would give such a close association with country incomes. As we show in Fig. 2 (B and C), similar associations between the ECI and income are also present in regional settings. Figure 2B shows that the ECI for U.K. local authorities is correlated with per-capita earnings, while Fig. 2C shows that the ECI for U.S. states is also correlated with state-level per-capita GDP. U.K. earnings data are sourced from the U.K. Office for National Statistics Annual Survey of Hours and Earning, and U.S. state-level per-capita GDP data are sourced from the U.S. Bureau of Economic Analysis.

#### Correspondence analysis

A second interpretation as a dimensionality reduction tool, which connects both the ECI and PCI, relates to correspondence analysis (*9*–*13*). Simple (multiple) correspondence analysis is a multivariate statistical method for analyzing relationships between two (more than two) categorical variables. It is frequently used to graphically visualize the association between row and column categories of a contingency table in a lower-dimensional space. If one treats the matrix *M* as a contingency table, then finding the eigenvectors corresponding to the largest eigenvalues of matrices $\tilde{M}$ and $\hat{M}$ (see Eqs. 5 and 7) is exactly equivalent to performing simple correspondence analysis on *M* (*12*, *14*).

An alternative technique to implement correspondence analysis is known as reciprocal averaging (*11*, *12*). This algorithm is equivalent to the method of reflections, which was originally proposed to calculate the ECI and PCI. As we show in the Supplementary Materials, a country’s ECI is the average of the PCI of products that it is competitive in [see also (*12*)].

**Fig. 3:**
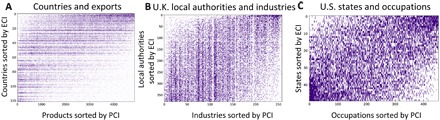
Ordering rows and columns of *M* by the ECI and PCI. In each matrix, rows are sorted by the ECI and columns are sorted by the PCI. (**A**) Country-product *M* matrix; (**B**) U.K. region-industry *M* matrix; (**C**) U.S. state-occupation *M* matrix.

Simple correspondence analysis arrives at orderings (given by the ECI and PCI) that maximize the correlation between two categorical variables (the rows and columns of *M*) (*12*). We provide an illustration of these orderings in Fig. 3, which shows the *M* matrix for countries and exports (Fig. 3A), U.K. local authorities and industries (Fig. 3B), and U.S. states and occupations (Fig. 3C). In all three cases, we sort the country, region, and state rows according to their corresponding ECI in ascending order. We also sort the export, industry, and occupation columns by their corresponding PCI in ascending order.

Putting together the insights from Figs. 2A and 3A, we can see that there is a systematic pattern of specialization in the export data. Richer countries with high ECI specialize in a similar set of high-PCI products, while poorer countries with low ECI tend to specialize in low-PCI products.

Inspecting the products at either end of the PCI spectrum allows us to infer information about the products that richer and poorer countries specialize in. As shown by Hausmann *et al.* (*2*), high-PCI products tend to relate to chemical and machinery exports that require technologically sophisticated know-how and advanced manufacturing processes, while low-PCI products tend to correspond to agricultural products or raw minerals.

The regional datasets also show similar patterns of specialization and correlation with earnings and income per capita, suggesting that richer (poorer) regions and states with high (low) ECI specialize in high-PCI (low-PCI) industries and occupations. In the Supplementary Materials, we show the top and bottom local authorities and U.S. states ranked by the ECI, as well as the top and bottom industries and occupations ranked by the PCI. In the United Kingdom, high-ECI (low-ECI) local authorities tend to be urban (rural) areas specialized in high-PCI (low-PCI) industries relating to financial and professional (agricultural and manufacturing) industries. We find similar results for the U.S. data.

### Revisiting previous interpretations of economic complexity

**Fig. 4:**
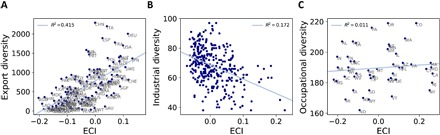
ECI versus diversity. Relationship between diversity and the ECI for data on (**A**) countries and exports, (**B**) U.K. regions and industries, and (**C**) U.S. states and occupations.

Previous interpretations of the ECI have tended to be cast in terms of diversity (*2*, *3*, *16*, *17*), although the ECI and diversity are mathematically orthogonal [see Eq. 24 and (*18*)]. However, in the country export data (see Fig. 4A) and in Chinese regional data (*16*), diversity and the ECI turn out to be positively correlated. Recall that orthogonality (having a zero dot product) does not imply zero correlation unless the mean of one of the variables is zero. Neither diversity nor the (unstandardized) ECI has zero means in these data. As we show in Fig. 4 (B and C), the empirical relationship between the ECI and diversity is different in the U.K. and U.S. regional data. Despite being positively correlated with regional per-capita earnings (Fig. 2, B and C), the ECI is negatively correlated with industrial diversity of U.K. local authorities and does not correlate with occupational diversity of U.S. states.

**Fig. 5:**
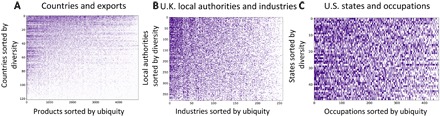
Ordering rows and columns of *M* by diversity and ubiquity. In each matrix, rows are sorted by diversity and columns are sorted by ubiquity. (**A**) Country-product *M* matrix; (**B**) U.K. region-industry *M* matrix; (**C**) U.S. state-occupation *M* matrix.

The mathematical orthogonality between the ECI and diversity indicates that these variables capture different information (*18*). In particular, previous work has shown that ordering the rows of matrix *M* by country diversity and the columns by product ubiquity reveals a triangular structure (see Fig. 5A) (*23*). This pattern indicates that more diverse countries tend to export less ubiquitous products, while less diverse countries tend to export more ubiquitous products, in contrast to traditional theories of comparative advantage (*23*).

**Fig. 6:**
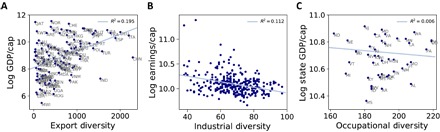
Diversity versus income per capita. (**A**) Relationship between diversity and log GDP per capita for data on countries and exports. (**B**) Relationship between diversity and log per-capita earnings for data on industrial employment concentrations in U.K. local authorities. (**C**) Relationship between diversity and log GDP per capita for data on occupational employment concentrations in U.S. states.

However, in both of our regional examples, diversity and ubiquity fail to be economically informative. As we can see in Fig. 5 (B and C), the diversity and ubiquity ordering of *M* matrices constructed from U.S. and U.K. regional data does not reveal a triangular structure. Moreover, as shown in Fig. 6, while country diversity is positively correlated with per-capita GDP in the export data (Fig. 6A), there is no positive correlation between diversity and per-capita earnings in the United Kingdom (Fig. 6B) or per-capita state-level GDP in the United States (Fig. 6C).

## DISCUSSION

This paper provides a number of mathematical interpretations of the ECI and PCI and shows how these interpretations offer useful insights into export and regional data. Our results also cast existing empirical findings in a new light. Previously, the success of the ECI in explaining variation in per-capita GDP and future growth rates across countries was thought to reflect the importance of accumulating a diverse set of productive capabilities (*2*, *3*, *23*). However, by making the difference between the ECI and diversity explicit, we can better understand the distinct roles that these variables play in the development process.

The relationship between diversification and development is well established in the economics literature. Countries tend to follow a U-shaped pattern, whereby they first diversify and then begin to specialize relatively late in the development process (*24*). This pattern aligns with other empirical studies that have described a positive association between export diversification and economic growth, which tends to be stronger for less developed countries (*25*–*27*).

In contrast to diversity, the application of the ECI and PCI to export data sheds light on specialization patterns across countries. High-PCI (low-PCI) products tend to be exported by richer (poorer), high-ECI (low-ECI) countries. As high-PCI (low-PCI) products tend to be more (less) technologically sophisticated (*2*), this finding underscores the importance of technological upgrading in the development process. While the relationship between technological capabilities and development has also received significant attention in economics (*28*–*30*), our interpretation of the ECI and PCI as dimensionality reduction tools offers a useful approach for analyzing the differences in the export baskets of low- and high-income countries.

The interpretations of the economic complexity measures discussed in this paper open a door for further applications of dimensionality reduction methods to other economic datasets. As we have shown with our illustration of the U.K. and U.S. employment data, the ECI and PCI reveal similar patterns of specialization across richer and poorer regions. Future work could readily extend the economic complexity measures to examine other economic networks, such as production networks constructed from country input-output data. Moreover, relationships between the ECI/PCI, diffusion maps (*14*, *31*), and simple correspondence analysis (*12*, *32*) (some of which are further discussed in the Supplementary Materials) suggest that new insights could be gleaned from applications of nonlinear diffusion maps and multiple correspondence analysis to economic data.

## MATERIALS AND METHODS

### Calculating the ECI for U.K. and U.S. regional employment data

#### U.K. local authorities and industries

Using data from the BRES, we constructed a binary region-industry matrix *W* on the basis of a region *r*’s location quotient (LQ) in industry *i*

$${\text{LQ}}_{ri}=\frac{e_{ri}/\Sigma_{i}e_{ri}}{\Sigma_{r}e_{ri}/\Sigma_{r}\Sigma_{i}e_{ri}}$$ (25)

where *e*_*ri*_ is the number of people employed in industry *i* in region *r* and *W*_*ri*_ = 1 if LQ_*ri*_ > 1 and LQ_*ri*_ = 0 otherwise. Note that Eq. 25 is analogous to Eq. 1. We then constructed a $\tilde{W}$ matrix from *W* in the same way as $\tilde{M}$ was constructed from *M* (Eq. 5). Last, we calculated the industry-based ECI for U.K. local authorities by finding the eigenvector associated with the second largest eigenvalue of $\tilde{W}$.

#### U.S. states and occupations

We applied the same methodology to calculate the occupation-based ECI for U.S. states. (We also found consistent results using data on U.S. states and industries.) Drawing on census data for the United States, which are available from the IPUMS (*22*), we constructed a state-occupation matrix using a state’s LQ in occupation *i*. We then computed the occupation-based ECI for U.S. states analogously to the industry-based ECI for U.K. local authorities.

## Supplementary Material

http://advances.sciencemag.org/cgi/content/full/5/1/eaau1705/DC1

## SUPPLEMENTARY MATERIALS

Supplementary material for this article is available at http://advances.sciencemag.org/cgi/content/full/5/1/eaau1705/DC1 Section S1. Diversity and degree equivalence Section S2. Relationship between the ECI and PCI Section S3. Interpretation of ECI as a diffusion map and relationships to correspondence analysis and kernel principal component analysis Section S4. ECI and PCI rankings for regional data Section S5. Eigengap heuristic analysis Section S6. Robustness of empirical results to alternative RCA thresholds Fig. S1. Application of diffusion map interpretation to country export data. Fig. S2. Top largest eigenvalues of the $\tilde{M}$ matrix for data on exports, U.K. regional industrial concentrations, and U.S. state occupational concentrations. Fig. S3. Robustness of ECI versus GDP/cap relationship to varying the RCA export threshold. Fig. S4. Country-product *M* matrix with rows sorted by the ECI and columns sorted by the PCI constructed using different RCA thresholds. Fig. S5. Robustness of ECI versus GDP/cap relationship to varying the RCA per-capita threshold. Table S1. Top and bottom 10 U.K. local authorities ranked by ECI. Table S2. Top and bottom 10 industries ranked by PCI. Table S3. Top and bottom 10 U.S. states ranked by ECI. Table S4. Top and bottom 10 occupations ranked by PCI. References (*33*–*35*)
