# Supplementary material for: Interpreting economic complexity
Source: Sci Adv. 2019 Jan 9;5(1):eaau1705. doi: 10.1126/sciadv.aau1705 (PMC6326748; doi:10.1126/sciadv.aau1705)
Supplement: http://advances.sciencemag.org/cgi/content/full/5/1/eaau1705/DC1 [file supp_5_1_eaau1705__index.html]

Science Advances | Science Advances

## Supplementary Materials

**This PDF file includes:**

- Section S1. Diversity and degree equivalence
- Section S2. Relationship between the ECI and PCI
- Section S3. Interpretation of ECI as a diffusion map and relationships to correspondence analysis and kernel principal component analysis
- Section S4. ECI and PCI rankings for regional data
- Section S5. Eigengap heuristic analysis
- Section S6. Robustness of empirical results to alternative RCA thresholds
- Fig. S1. Application of diffusion map interpretation to country export data.
- Fig. S2. Top largest eigenvalues of the M~ matrix for data on exports, U.K. regional industrial concentrations, and U.S. state occupational concentrations.
- Fig. S3. Robustness of ECI versus GDP/cap relationship to varying the RCA export threshold.
- Fig. S4. Country-product *M* matrix with rows sorted by the ECI and columns sorted by the PCI constructed using different RCA thresholds.
- Fig. S5. Robustness of ECI versus GDP/cap relationship to varying the RCA per-capita threshold.
- Table S1. Top and bottom 10 U.K. local authorities ranked by ECI.
- Table S2. Top and bottom 10 industries ranked by PCI.
- Table S3. Top and bottom 10 U.S. states ranked by ECI.
- Table S4. Top and bottom 10 occupations ranked by PCI.
- References (*33*–*35*)

Download PDF

**Files in this Data Supplement:**

- Adobe PDF - aau1705\_SM.pdf
