# Supplementary material for: Interpreting economic complexity
Source: Sci Adv. 2019 Jan 9;5(1):eaau1705. doi: 10.1126/sciadv.aau1705 (PMC6326748; doi:10.1126/sciadv.aau1705)
Supplement: http://advances.sciencemag.org/cgi/content/full/5/1/eaau1705/DC1 [file aau1705_SM.pdf]

Science Advances
AAAS

advances.sciencemag.org/cgi/content/full/5/1/eaau1705/DC1

Supplementary Materials for

Interpreting economic complexity

Penny Mealy*, J. Doyne Farmer, Alexander Teytelboym

*Corresponding author. Email: penny.mealy@inet.ox.ac.uk

Published 9 January 2019, *Sci. Adv.* **5**, eaau1705 (2019)
DOI: 10.1126/sciadv.aau1705

This PDF file includes:

Section S1. Diversity and degree equivalence
Section S2. Relationship between the ECI and PCI
Section S3. Interpretation of ECI as a diffusion map and relationships to correspondence analysis and kernel principal component analysis
Section S4. ECI and PCI rankings for regional data
Section S5. Eigengap heuristic analysis
Section S6. Robustness of empirical results to alternative RCA thresholds
Fig. S1. Application of diffusion map interpretation to country export data.
Fig. S2. Top largest eigenvalues of the $\widetilde{M}$ matrix for data on exports, U.K. regional industrial concentrations, and U.S. state occupational concentrations.
Fig. S3. Robustness of ECI versus GDP/cap relationship to varying the RCA export threshold.
Fig. S4. Country-product M matrix with rows sorted by the ECI and columns sorted by the PCI constructed using different RCA thresholds.
Fig. S5. Robustness of ECI versus GDP/cap relationship to varying the RCA per-capita threshold.
Table S1. Top and bottom 10 U.K. local authorities ranked by ECI.
Table S2. Top and bottom 10 industries ranked by PCI.
Table S3. Top and bottom 10 U.S. states ranked by ECI.
Table S4. Top and bottom 10 occupations ranked by PCI.
References (*33–35*)

Section S1. Diversity and degree equivalence

Recall that diversity is defined as

$$k_c^{(0)} = \sum_p M_{cp} \tag{1}$$

and the degree of a node in a graph defined by a similarity matrix S is

$$d_i = \sum_j S_{ij} \tag{2}$$

We want to show that these are equivalent. We defined $S = MU^{-1}M'$. Note that $U^{-1}M'$ is row-stochastic and $D^{-1}M$ is also row-stochastic. Therefore, any row of $\widetilde{M} = D^{-1}MU^{-1}M'$ adds up to 1 and hence every row i of $MU^{-1}M'$ must add up to D_{ii}.

Section S2. Relationship between the ECI and PCI

Proposition 1 (see Definition 2 on p. 342 in Hill [12]). *A country's ECI is equal to the average PCI of products that the country has revealed comparative advantage in.*

Proof. Recall that

$$\widetilde{M} = D^{-1}MU^{-1}M' \tag{3}$$

The ECI is one of the solutions $\widetilde{y}$ to the following eigensystem

$$\widetilde{M}\widetilde{y} = \widetilde{\lambda}\widetilde{y} \tag{4}$$

To calculate the PCI for all products, we are interested in the second eigenvector of the matrix $\widehat{M}$, which is given by

$$\widehat{M} = U^{-1} M' D^{-1} M \tag{5}$$

Hence, PCI is one of the solutions $\widehat{y}$ to the following eigensystem:

$$\widehat{M}\widehat{y} = \widehat{\lambda}\widehat{y} \tag{6}$$

To prove the proposition, take Eq. (4), the eigensystem for ECI, and substitute in Eq. (3)

$$D^{-1} M U^{-1} M' \widetilde{y} = \widetilde{\lambda}\widetilde{y} \tag{7}$$

$$M^{-1} D D^{-1} M U^{-1} M' \widetilde{y} = \widetilde{\lambda} M^{-1} D \widetilde{y} \tag{8}$$

$$U^{-1} M' \widetilde{y} = \widetilde{\lambda} M^{-1} D \widetilde{y} \tag{9}$$

which is equivalent to the eigensystem for PCI

$$U^{-1} M' D^{-1} M \widehat{y} = \widehat{\lambda}\widehat{y} \tag{10}$$

for

$$\widetilde{y} = D^{-1} M \widehat{y} \tag{11}$$

as required. □

Therefore, the ECI can be immediately obtained from the PCI by using M.

Section S3. Interpretation of ECI as a diffusion map and relationships to correspondence analysis and kernel principal component analysis

A *diffusion map* is a dimensionality reduction method that generates representations of complex data sets in a lower-dimensional Euclidean space by iterating the Markov matrix associated with the data [8, 31]. Since $\widetilde{M}$ can be seen as a Markov transition matrix (see section "The ECI and PCI" in the main paper), the ECI can also be used to construct a *basic diffusion map* that indicates how a random walker beginning at a particular node (or Markov chain "state") will move through the system [14].

For example, if we let the nodes in graph S represent states in a Markov transition matrix, the probability that a random walk beginning in state i reaches state j in the next step is given by $\widetilde{M}_{ij}$. Now consider two random walks beginning in states i and j. How "far" the random walks are from each other at time t tells us something about the similarity of nodes i and j in graph S. Let vector $x_i(t)$ denote the probability distribution over states reached at time t by a random walk beginning in state i. Then define the *diffusion map distance* to be proportional to

$$(x_i(t) - x_j(t))' D^{-1}((x_i(t) - x_j(t)) \tag{12}$$

Each states at time t can be represented as a point in an n-dimensional Euclidean space with coordinates

$$(|\lambda_2^t| y_i^{[2]}, |\lambda_3^t| y_i^{[3]}, \ldots, \lambda_n^t | y_i^{[n]})$$

where λ_j is the eigenvalue associated with the jth largest eigenvector $\widetilde{M}$ and $y_i^{[n]}$ is the ith entry of the nth largest eigenvector of $\widetilde{M}$ [14]. The distance between the points is precisely the diffusion map distance.

In fig. S1, we apply the diffusion map to country export data. By using the second and third coordinates of the diffusion map, we visualize countries in a two-dimensional plane at different t. Since the second largest eigenvalue is dominant, we rescale the axis by its value. As t goes to infinity, the diffusion map distance captures the distance between the stationary probabilities of states in the random walk and is well approximated by the second-largest eigenvector of $\widetilde{M}$ i.e. ECI [8].

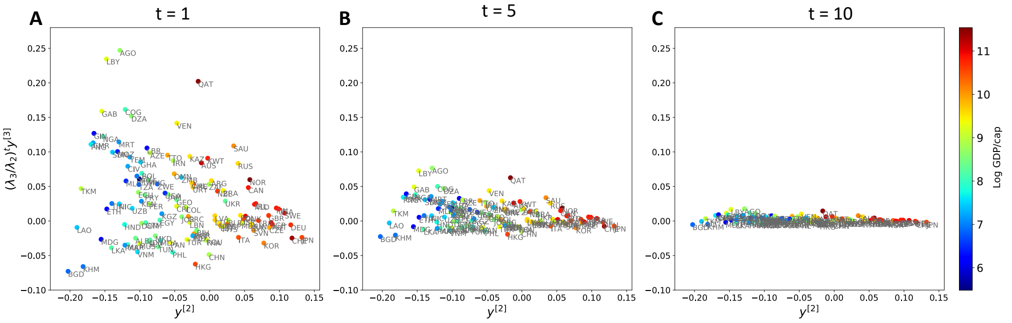

Fig. S1. Application of diffusion map interpretation to country export data.

There is also an equivalence between the *Ncut* criterion and *correspondence analysis* (CA) [32]. Simple (multiple) CA is a classic tool in multivariate analysis that studies relationships between two (two or more) categorical variables, such as countries and products, via singular value decomposition [9, 10, 12, 13]. In this setting, the similarity matrix S represents the *Pearson correlation matrix*. Performing simple correspondence analysis is equivalent to computing the basic diffusion map when $t = 1$ [14].

Finally, diffusion maps are also related to *kernel Principal Component Analysis* (PCA). Define

$$K(t) = \widetilde{M}^t D^{-1} \widetilde{M}'^t \tag{13}$$

which is a symmetric, positive-definite matrix known as the *diffusion map kernel*. Denote $w^{[n]}$ to be an eigenvector of K associated with μ_n, the nth largest eigenvalue. Each state at time t can be represented in an n-dimensional Euclidean space with coordinates

$$(\sqrt{\mu_1} w_i^{[1]}, \sqrt{\mu_2} w_i^{[2]}, \ldots, \sqrt{\mu_n} w_i^{[n]}) \tag{14}$$

This is not only a vector representation of each in the principal component space, but also the distance between the points is exactly the diffusion map distance.

A clear summary of relationships between different spectral methods for computing a low-dimensional embedding of undirected weighted graphs can be found in [34, Table 10.1, p. 439].

Section S4. ECI and PCI rankings for regional data

In this section, we show the top and bottom ECI and PCI rankings for UK local authorities (table S1), UK industries (table S2), US states (table S3) and US occupations (table S4).

Table S1. Top and bottom 10 U.K. local authorities ranked by ECI.

ECI Rank	Local Authority	ECI Rank	Local Authority
1	Tower Hamlets	371	Angus
2	City of London	372	Aberdeenshire
3	Islington	373	Allerdale
4	Westminster	374	Erewash
5	Southwark	375	Ribble Valley
6	Camden	376	Kirklees
7	Hammersmith and Fulham	377	Barnsley
8	Kensington and Chelsea	378	Dumfries and Galloway
9	Hackney	379	Neath Port Talbot
10	Cambridge	380	North Lincolnshire

Table S2. Top and bottom 10 industries ranked by PCI.

PCI Rank	Industry	PCI Rank	Industry
1	Reinsurance	249	Manufacture of articles of fur
2	Fund management activities	250	Manufacture of other products of first processing of steel
3	Television programming and broadcasting activities	251	Manufacture of basic iron and steel and of ferro-alloys
4	Trusts, funds and similar financial entities	252	Processing and preserving of meat and production of meat products
5	Manufacture of magnetic and optical media	253	Manufacture of refractory products
6	Legal activities	254	Manufacture of cement, lime and plaster
7	Activities auxiliary to financial services	255	Preparation and spinning of textile fibres
8	Market research and public opinion polling	256	Weaving of textiles
9	Accounting, bookkeeping and auditing activities	257	Mining of hard coal
10	Advertising	258	Manufacture of coke oven products

Table S3. Top and bottom 10 U.S. states ranked by ECI.

ECI Rank	US State	ECI Rank	US State
1	California	41	Wisconsin
2	New Jersey	42	Ohio
3	Maryland	43	Tennessee
4	Massachusetts	44	Michigan
5	New York	45	South Carolina
6	Connecticut	46	Alabama
7	Colorado	47	Arkansas
8	Virginia	48	Indiana
9	Washington	49	Mississippi
10	Arizona	50	Kentucky

Table S4. Top and bottom 10 occupations ranked by PCI.

PCI Rank	Occupation	PCI Rank	Occupation
1	Lawyers, and judges, magistrates, and other judicial workers	444	Metal workers and plastic workers, nec
2	Actors, Producers, and Directors	445	Laborers and Freight, Stock, and Material Movers, Hand
3	Editors, News Analysts, Reporters, and Correspondents	446	Assemblers and Fabricators, nec
4	Software Developers, Applications and Systems Software	447	Other production workers including semiconductor processors
5	Financial Analysts	448	Welding, Soldering, and Brazing Workers
6	Accountants and Auditors	449	Millwrights
7	Securities, Commodities, and Financial Services Sales Agents	450	Driver/Sales Workers and Truck Drivers
8	Computer Scientists and Systems Analysts	451	Grinding, Lapping, Polishing, and Buffing Machine Tool Setters
9	Personal Financial Advisors	452	Cutting, Punching, and Press Machine Setters
10	Managers, nec (including Postmasters)	453	Extruding, Forming, Pressing, and Compacting Machine Setters

Section S5. Eigengap heuristic analysis

In section "Applying the spectral clustering interpretation to economic data" in the main paper, we showed that similarity networks constructed from the export and regional datasets did not partition well into two clusters. Here we analyse what is known as the *eigengap heuristic*, which is a standard methodology used in spectral clustering analysis for determining the number of clusters present in the graph [35].

The eigengap heuristic involves choosing the number of k clusters such that the largest eigenvalues $\lambda_1, ..., \lambda_k$ of $\widetilde{M}$ are large, while λ_{k+1} is relatively small. In fig. S2, we show the largest six eigenvalues of the $\widetilde{M}$ matrix calculated for data on exports, UK regional industrial concentrations, and US state occupational concentrations respectively. In all three cases, the largest gap occurs between the first and second eigenvalue ($|\lambda_2 - \lambda_1|$). According to the eigengap heuristic, this suggests that from a spectral clustering perspective

the graphs considered in this paper are likely to only contain one cluster. However, it is also important to note that the eigengap heuristic usually only works well if the data contains well-pronounced clusters - which is not the case here.

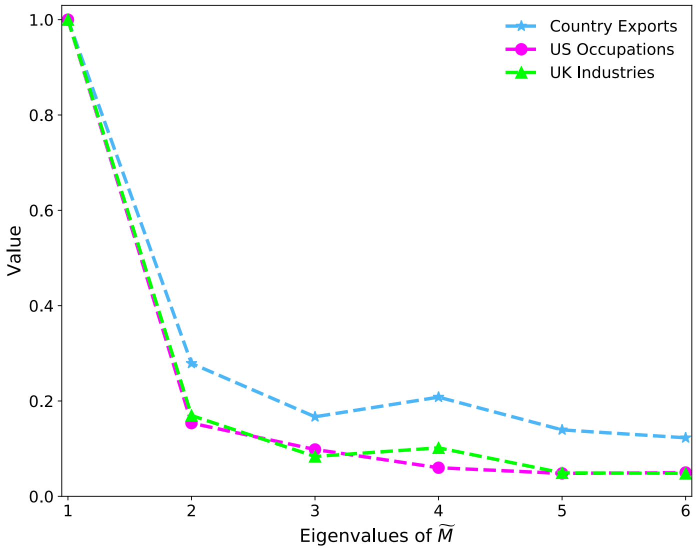

Fig. S2. Top largest eigenvalues of the $\widetilde{M}$ matrix for data on exports, U.K. regional industrial concentrations, and U.S. state occupational concentrations.

Section S6. Robustness of empirical results to alternative RCA thresholds

In principle, the use of the RCA measure to calculate the binary M matrix can be particularly sensitive to the chosen threshold above which a country is considered to have a revealed comparative advantage in a product. For the empirical results shown in the main paper we have followed the most common approach and used a threshold of 1. While the choice of threshold will have no bearing on the mathematical interpretation of the ECI and PCI, in this section we show to the extent to which empirical results for the country-export data are influenced by different RCA thresholds.

In Panel A of fig. S3, we show how the empirical correlation between the ECI and per capita GDP change for different RCA thresholds. Correlations are highest between thresholds of 0.5 and 2. Panel B shows the correlation between the ECI and country diversity for different RCA thresholds.

When the RCA threshold is zero, there are some products that are competitively exported by all countries. This means that the multiplicity of the largest eigenvalue is greater than one. In this case, since the eigenvector corresponding to the largest eigenvalue is proportional to diversity, the eigenvector corresponding to the second-largest eigenvalue is also proportional to diversity. Therefore, when the RCA threshold is zero, there is a perfect correlation between ECI and diversity.

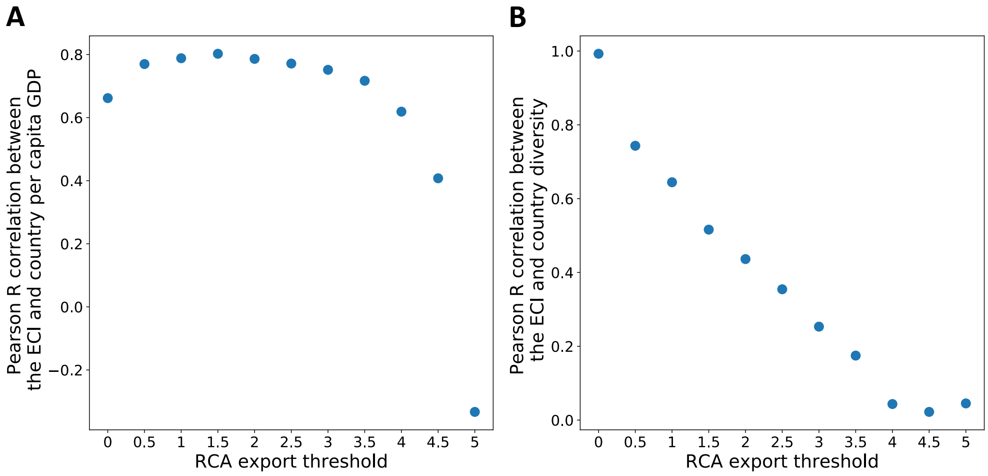

Fig. S3. Robustness of ECI versus GDP/cap relationship to varying the RCA export threshold. **Panel A)** Pearson correlation between the ECI and country per capita GDP for different RCA export thresholds. **Panel B)** Pearson correlation between the ECI and diversity for different RCA export thresholds.

Figure S4 examines how the pattern of specialization revealed by the ECI and PCI changes for different RCA thresholds. Here we compare binary M matrices, each sorted by ECI and PCI, using RCA thresholds of 0.5, 1 and 2. The pattern becomes more triangular for the lower RCA threshold (Panel A), largely because the ECI ordering is becoming closer to the ordering given by diversity (see Panel B of fig. S3). The higher RCA threshold (Panel

C) shows a similar pattern of specialization to the original pattern shown in Panel B.

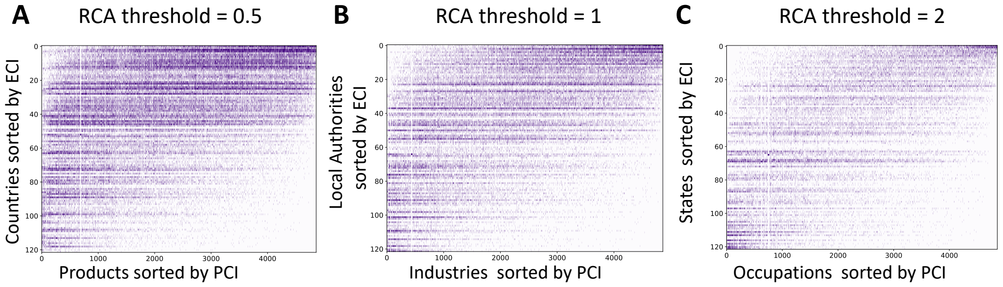

Fig. S4. Country-product M matrix with rows sorted by the ECI and columns sorted by the PCI constructed using different RCA thresholds. **Panel A)** RCA threshold = 0.5; **Panel B)** RCA threshold = 1; **Panel C)** RCA threshold = 2;

We also follow the approach taken in [33] and examine how the correlation between the ECI, per capita GDP and diversity change using a "per capita" version of RCA (RCA_POP), given by

$$RCA\_POP_{cp} = \frac{x_{cp}/n_c}{\sum_c x_{cp}/\sum_c n_c} \tag{15}$$

where x_{cp} is country c's exports of product p, n_c is the population of country c and $M_{cp} = 0$ otherwise.

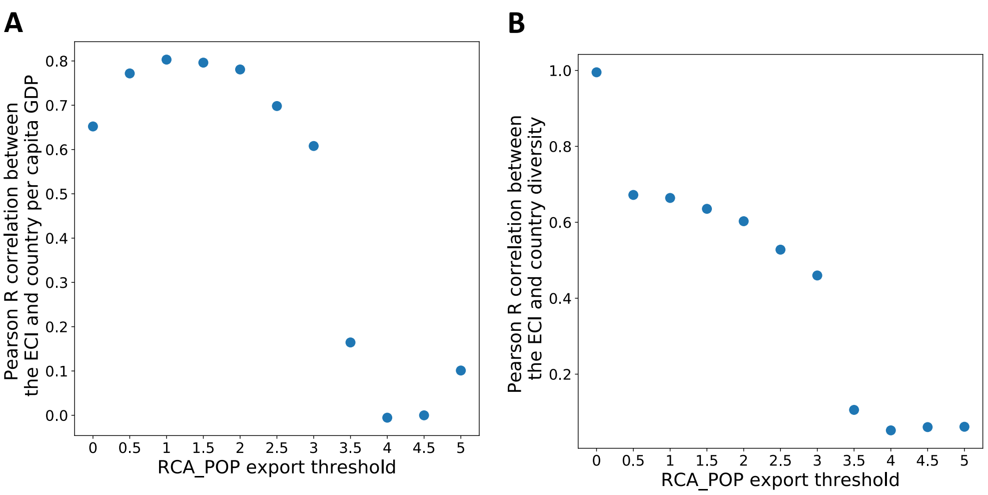

Fig. S5. Robustness of ECI versus GDP/cap relationship to varying the RCA per-capita threshold. **Panel A)** Pearson correlation between the ECI and country per capita GDP for different per capita RCA thresholds. **Panel B)** Pearson correlation between the ECI and diversity for different per capita RCA thresholds.

Our results suggest that regardless of whether the per capita or original RCA version is applied, a threshold of 1 gives a strong correlation to per capita GDP. Moreover, the correlation between diversity and the ECI decreases as the RCA threshold is increased.
